# Dual-task testing in adults with persistent postconcussive symptoms: a scoping review with conceptual mapping approach

**DOI:** 10.1007/s00221-026-07392-w

**Published:** 2026-09-06

**Authors:** Daniel Büchel, Stefan J. Blaschke, Lisa Krampe, Victoria Prockl, Stefanie Klatt, Bettina Wollesen

**Affiliations:** 1 Institute of Movement Therapy and Movement-Oriented Prevention and Rehabilitation, German Sports University Cologne, Cologne, Germany; 2 Faculty of Medicine and University Hospital, Department of Neurology, University of Cologne, Cologne, Germany; 3 Institute of Exercise Training and Sport Informatics, German Sport University Cologne, Cologne, Germany

**Keywords:** Brain injury, Dual-task, Symptoms, Chronic concussion, Gait, Balance, Cognition

## Abstract

**Background:**

Persistent postconcussive symptoms (PPCS) affect approximately 30% of individuals following mild traumatic brain injury, resulting in prolonged sensorimotor and cognitive disruptions. While traditional assessments focus on isolated subfunctions, they often fail to capture the complex integration required for daily activities. This scoping review aimed to summarize the evidence on dual-task testing in PPCS, identify frequently used subtasks, and map these paradigms to clinical symptom clusters.

**Methods:**

A systematic search of MEDLINE, CINAHL, and EMBASE was conducted. Studies were included if they involved adults (18–59 years) with persistent symptoms (> 14 days post-injury) performing simultaneous motor and cognitive tasks compared to single-task conditions. Methodological quality was assessed using a modified Downs and Black checklist. Conceptual mapping was employed to visualize the co-occurrence of symptoms and DT subtasks.

**Results:**

Eight studies (*N* = 197) met the eligibility criteria. Seven studies reported significant cognitive-motor interference (CMI) in PPCS cohorts compared to healthy controls. Gait was the primary motor task investigated, while cognitive tasks focused on working memory (e.g., arithmetic) and inhibition (e.g., Stroop). Conceptual mapping revealed that general symptom burden and anxiety were central hubs linked to gait and executive function tasks. However, specific clusters like fatigue, headache, and ocular dysfunction were notably underrepresented.

**Significance:**

Dual-task assessments, particularly combining gait with executive function tasks, are sensitive markers for functional deficits in PPCS. Future research should standardize protocols, include cognitive response times to capture speed-accuracy trade-offs, and utilize neurophysiological applications such as electroencephalography to better understand the underlying neural resource reallocation.

**Supplementary Information:**

The online version contains supplementary material available at https://doi.org/10.1007/s00221-026-07392-w.

## Introduction

Traumatic brain injuries (TBIs) result from sudden, direct or indirect impacts to the head, leading to a significant disruption of sensorimotor and cognitive control systems (Maas et al. 2022). Depending on the initial clinical presentation, about 90% of cases are labeled as “mild” TBI (mTBI), but nonetheless often accompanied by diffuse neuropsychological symptoms termed “concussion”. Concussions are characterized by a complex constellation of symptoms, including ocular, vestibular, and cognitive dysfunctions, fatigue, headache, and anxiety (Harmon et al. 2019).

While most symptoms resolve within 10–14 days, a relevant subset (~ 30%) develops lasting impairments, in some cases for months after trauma, termed persistent postconcussive symptoms (PPCS). PPCS describes an insufficiently understood phenotype with secondary, undulating increase of the above-mentioned concussive symptoms that potentially persist over weeks to months (Mittenberg and Strauman 2000). While persistence of symptoms is often attributable to previous, often unrecognized, impairments in certain functional domains prior to injury or comorbidities (McIntosh et al. 2025), it can manifest as an inability to navigate complex everyday environments, highlighting a critical need for objective monitoring of functional and locomotor recovery to facilitate targeted treatment (Broglio et al. 2019). However, no single biomarker, clinical parameter, or combination of assessment tools has yet demonstrated sufficient reliability to definitively diagnose or monitor persistent post-concussion symptoms. This diagnostic limitationcontributes to clinical uncertainty and may increase the risk of misdiagnosis, potentially resulting in the underrecognition of treatable impairments. Therefore, the development and implementation of more objective diagnostic measures are essential to enable standardized tracking of functional recovery across clinical and professional settings (Broglio et al. 2019). Crucially, persistent symptoms as early as 14 days after concussion have recently been shown to significantly predict the impact on everyday life three to six months later (Eagle et al. 2025). This finding highlights the value of the subacute period as a critical timeframe for early interventions.

Typical objective assessments test ocular, vestibular, and cognitive subfunctions affected by concussions in an isolated manner. For instance, the Immediate Postconcussion Assessment and Cognitive Testing (ImPACT) programme (Mayers and Redick 2012) is a computerized test battery focusing on isolated cognitive domains such as verbal recognition memory, visual working memory, or visual processing speed. However, complex real-life situations like navigation in traffic or sports environments require the integration of ocular, vestibular, and cognitive processes into movement (DeAngelis and Angelaki 2012). In such situations, performance detriments in the sub-tasks are expected, as multiple sub-processes (e.g., visual screening, decision making, and movement initiation) compete for similar cognitive resources, resulting in performance bottlenecks (Ruthruff et al. 2001). For instance, the frontoparietal network plays a crucial role in structural interference, largely driven by the fact that motor control and executive functions utilize shared neural pathways (Marek and Dosenbach 2018). The competition for shared resources results in a phenomenon referred to as cognitive-motor interference (CMI). CMI describes a performance reduction when doing a motor and cognitive task at the same time and seems to rely on the individual attentional resources available (Leone et al. 2017). Given the acute to chronic impairments of cognitive and motor resources in PPCS, individuals appear particularly susceptible to CMI when compared to healthy individuals (Fino et al. 2018). Therefore, CMI might serve as a promising objective indicator of underlying resource competition and cognitive-motor integration, though it is important to note that statistically significant laboratory interference does not always directly translate to everyday functional impairment.

CMI can be assessed through dual-task paradigms which require the parallel execution of a motor and cognitive subtask and provide valuable insights into the functional capacity of individuals through the quantification of dual-task costs (McCulloch 2007). These measure the absolute or relative performance loss in the motor and cognitive subtasks when doing two tasks in parallel and serve as an index of CMI. In a recent study by Bryk et al. (2023), Individuals with PPCS demonstrated higher dual-task costs by means of reduced gait speed and response accuracy when walking and performing a working memory task in parallel. Although dual-tasks seem a promising approach to expose increased CMI in individuals with PPCS, the optimal design of such paradigms remains unclear, particularly with respect to symptom-specific demands as outlined by Harmon et al. (2019). For instance, individuals with vestibular dysfunction, such as bilateral vestibulopathy and unilateral vestibular dysfunction, often are unable to perform gait and working memory tasks in parallel due to nausea (Nascimbeni et al. 2010). On the other hand, patients suffering from chronic fatigue maintained adequate performance in static postural and working memory tasks (Rasouli et al. 2018). Thus, the interplay between PPCS symptoms and DT subtasks implies a careful synthesis of the literature to optimize dual-task experiment design. While previous systematic reviews have already summarized DT research in individuals with acute concussion(Büttner et al. 2020; DuPlessis et al. 2023; Fino et al. 2018; Mitchell and Cronin 2023), little is known about the subgroup of PPCS. Concept maps offer a research tool to organize existing knowledge and allow for a schematic overview of the current state of evidence (Novak and Cañas 2006). To this end, a concept mapping of the actual literature on DT and PPCS populations allows linking of dual-task experiments with existing schemes such as the concussion symptom flower by Harmon et al. (2019) in one comprehensive scheme. The symptom flower by Harmon et al. (2019) was chosen since it displays clinical profiles in a comprehensive, overlapping manner, allowing for a more abstract perspective on the highly individual concussion symptom profiles which are distinguished in clinical practice. Due to its link to clinical assessment, the elements of the symptom cluster are considered in the common concussion assessments (Feddermann-Demont et al. 2017), so that the flower comes with substantial ecological validity.

Therefore, the present review aims to summarize the current evidence on dual-task investigations in patients with persisting symptoms after the concussion and map the dual-task paradigms investigated with special regard to the specific clinical subdomains affected. First, we aimed to summarize the current evidence on dual-task testing beyond the acute phase to monitor lasting symptoms after concussion in either the subacute or persistent (i.e., PPCS) phase. Second, we aimed to describe which motor and cognitive tasks are most frequently used in dual-task investigations involving adults with PPCS. Third, we aimed to map how identified dual-task subtasks align with the symptom clusters defined by Harmon et al. (2019). Rather than testing specific a priori hypotheses, this exploratory mapping was performed to evaluate if current research indicates that certain symptom profiles associate with specific dual-task paradigms. Therefore, the concept map aims to facilitate the linking of dual-task subtasks with specific clinical domains. Based on the conceptual mapping, current research gaps can be exposed to guide the development of tailored, symptom-specific dual-task protocols in PPCS cohorts.

## Materials and methods

### Protocol structure and registration

This study was planned as a systematic review and was registered in advance of the literature search with the International Prospective Register of Systematic Reviews (PROSPERO; registration number: CRD420251057670). However, the limited number and observed heterogeneity of identified studies required to conduct a scoping review to generate a broad overview of dual-task practices in clinical and applied research by mapping the associations between PPCS symptoms and dual-task setups rather than answering a specific research question (Munn et al. 2018). Therefore, the present review adheres to the Preferred Reporting Items for Systematic Reviews and Meta-Analysis Extension for Scoping Reviews (PRISMA-ScR) guidelines (Tricco et al. 2018).

## Eligibility criteria

We established eligibility criteria based on the PEO framework, which defines *Population*, *Exposure*, and *Outcomes* as key elements to structure the evidence on dual-task research in PPCS (Hosseini et al. 2024). Furthermore, we added the element „Study Design” to emphasize the focus on original research conducted with PPCS populations.

For the element *Population*, we included studies involving adults aged 18–59 years, which encompasses both young adults (18–39 years) and middle-aged adults (40–59 years), consistent with classifications proposed (Kowall et al. 2023). Accordingly, we excluded studies focusing on participants younger than 18 years (children and adolescents) or older than 60 years (older adults). Further, eligible studies must involve participants who experienced a concussion and report symptoms beyond the acute phase, i.e., lasting longer than 14 days after concussion, that have a higher likelihood of developing lasting impairments over months (Eagle et al. 2025). This way, we aimed at mapping specific DT paradigms in the subacute and persistent (i.e., PPCS) phase after concussion. Accordingly, studies testing individuals within the first two weeks after concussion without follow-up were not considered eligible. In order to consider symptom clusters, included studies must also refer to at least one validated symptom inventory. Studies focusing on self-reported concussion history that did not utilize a validated inventory were excluded from this review.

For the element *Exposure*, we included studies that assessed dual-task paradigms, specifically those requiring the simultaneous and parallel performance of a motor and a cognitive task. To be eligible, the dual-task paradigm must allow the evaluation of CMI by comparing dual-task performance to single-task (ST) performance. Accordingly, we excluded studies that used dual-task paradigms that combined two motor tasks or two cognitive tasks, as well as those involving incorporated dual-task activities, such as Speedcourt-based training, reactive agility tasks, or other complex multimodal tasks that do not allow for a clear separation of cognitive and motor demands (for specification, see (Torre and Temprado 2021).

For the element *Outcome*, we included studies that quantified motor behavior and cognitive function. Motor behavior can be described based on quantitative parameters such as gait metrics (e.g., cadence, step length, step width), postural control metrics (e.g., area of sway, sway velocity), or task-specific performance outcomes (task time, task accuracy or errors). For cognitive function, studies with a specific focus on domains like working memory, inhibition, or cognitive flexibility were considered. Outcomes were categorized into time-based (reaction times) and performance-based (accuracy/errors) measures. Although data on cognitive performance are often missing in serial subtraction tasks, we included such studies due to their prominence in dual-task research and report outcomes as not reported (Wollesen et al. 2019). Further, only studies deriving CMI based on the comparison of single- and dual-task performance were included. Here, we considered studies that investigated CMI in the motor domain, cognitive domain, as well as both domains.

For the element *Study design*, we included original research, including cross-sectional studies, cohort studies, case-control studies, observational studies, intervention studies, and randomized controlled trials (RCTs). We excluded single-case studies, books, student theses, and review articles.

## Information sources and search strategies

Three databases (MEDLINE, CINAHL, and EMBASE) were searched and integrated on July 20 2025. To ensure a broad review of the available literature, no restrictions on publication date were applied. No filters or limits were used in the search. All references were directly imported to Rayyan AI (Ouzzani et al. 2016) for deduplication and blinded screening. According to the previously mentioned PEO principle, the combined search term can be extracted from the keywords presented in Appendix I. All three concepts were combined through the Boolean operator “AND”.

## Study selection and data extraction process

**Fig. 1:**
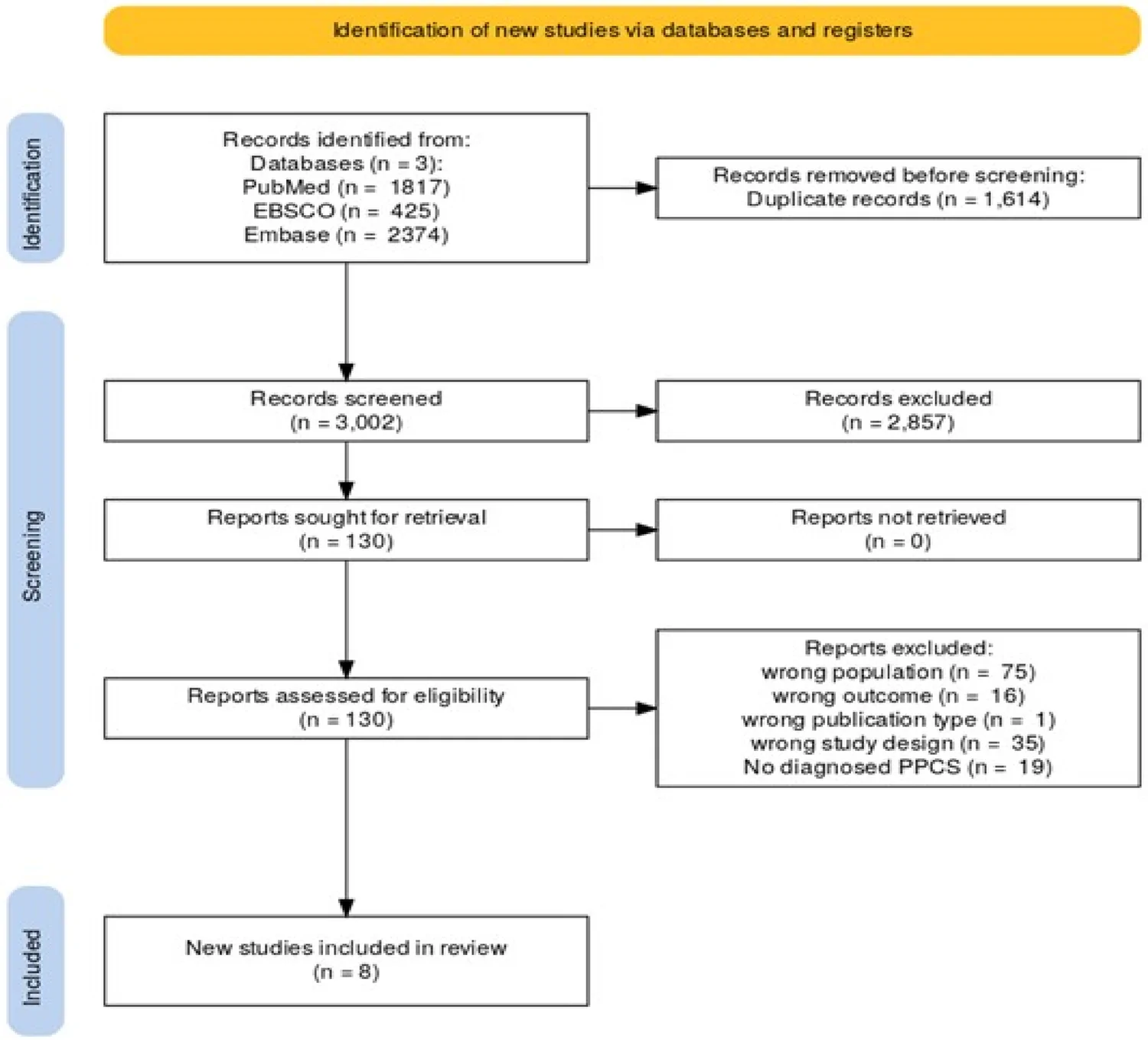
PRISMA-ScR flow diagram for the scoping review study selection process

After import into Rayyan AI, duplicates were identified and removed before the start of the title and abstract screening guided by the PEO framework. DB, SB, LK, VP, SK, and BW independently screened records for inclusion, and each author screened a third of the original search hits. Each hit was screened by two researchers who were blinded to each other’s decisions. For articles marked eligible after title and abstract screening, full-texts were uploaded into Rayyan. Both title and abstract screening as well as full-text screening were performed following the same procedure. Disagreement between individual judgments was resolved by a third reviewer. The selection process followed the recommendations of the PRISMA checklist and is reported in the PRISMA chart in Fig. 1.

## Data extraction

Data extraction was independently conducted by DB and SB using a standardized Excel spreadsheet. The data extraction sheet was piloted on a sample of studies to ensure consistency and clarity. Data from each included study were independently extracted by DB and SB and collated afterwards. Any discrepancies during data extraction were discussed with a third reviewer to reach consensus. The following information were charted: (i) Study metadata: author, year of publication; (ii) Sample demographics: number of participants, Number of cases, sample specification (athletes, students, military, employees, etc.), number of female cases, age range; (iii) PPCS symptoms: TBI specification (severity, time from TBI event), PPCS symptom specification: generic (not defined) or specific based on symptom inventory; (iv) dual-task specifications: Description of the motor and cognitive tasks performed, description of the outcomes extracted. For motor tasks, gait parameters and postural stability outcomes were considered; for cognitive tasks, accuracy/ error rates and time measures (response times) were considered; (v) Study Findings/ Results: Description of main findings, reporting of statistical outcomes. In case data were missing, the corresponding authors of the articles were contacted and asked for additional data reporting according to the Cochrane guidelines (Young and Hopewell 2011).

### Critical appraisal

The methodological quality of included articles was evaluated using a modified version of the Downs and Black (1998) questionnaire (Downs and Black 1998; Wunderlich et al. 2024). Accordingly, DB and SB assessed the methodological quality of the included studies independently. The questionnaire evaluated the following domains: (i) Study Reporting, (ii) External Validity, (iii) Internal Validity, and (iv) Power. The quality of the included studies was tabulated and considered when interpreting the results of the review. Any discrepancies in quality assessment were resolved through discussions with a third reviewer to provide a final decision when no consensus was reached.

## Synthesis of results

The purpose of our scoping review was to summarize the current evidence on dual-task testing in PPCS populations. Therefore, we aimed to describe the characteristics of existing experimental studies. Therefore, all articles and their characteristics were systematically synthesized by DB and SB with a focus on PPCS population demographics, a thorough description of the dual-task setups, and the findings of the studies.

## Concept mapping

Concept mapping was used to identify structural relationships between symptom domains and task characteristics that are not apparent from descriptive synthesis alone. Therefore, both pre-defined and post-hoc categorized concepts were extracted for the included studies. First, the PPCS symptom clusters according to Harmon et al. (2019) served as pre-defined concepts representative of affected clinical subdomains. Further, the subtasks of the identified dual-task studies were categorized and served as post-hoc concepts based on the data extraction table. For the generation of the co-occurrence map, DB and SB manually extracted the data and binary-coded (1 = present; 0 = absent) concept occurrence for each included study, resulting in a binary matrix listing studies as rows and concepts as columns. This binary table was transferred into a concept co-occurrence matrix, which formed the base for the concept mapping using customized scripts for MATLAB (R2025a, The MathWorks). Occurrences of symptom clusters were assigned based on the symptom inventories used in the included articles. If the identified symptom inventory (e.g., the Beck’s Depression Index) was specifically targeting one symptom cluster (e.g., anxiety), we assigned an occurrence of the symptom „anxiety“ for a given study. Overall symptom burden was noted if general symptom inventories (e.g., Neurobehavioral Symptom Inventory and the Rivermead Post-Concussion Symptoms Questionnaire) were used without an explicit sub-analysis of symptom subscales. In the resulting map, each concept represents a node and the intensity of edges between two nodes represents the frequency with which two concepts co-occur across the investigated sample of studies. Our mapping protocol explicitly excluded intra-domain connections (e.g., motor-to-motor). Consequently, edges were solely generated to illustrate the co-occurrence between distinct modules (i.e., linking motor tasks, cognitive tasks, and symptom domains). All scripts are available upon reasonable request.

## Results

### Study selection

The initial search generated 4616 articles, of which 1614 were excluded as duplicates (Fig. 1). Based on the predefined PEO criteria, 75 were excluded because they investigated acute concussions. 19 studies were excluded because persistence of clinical symptoms was not reported. 35 studies were excluded becausethey did not assess a dual-task with a combination of motor and cognitive subtasks. For the final review, a total of eight articles were identified as eligible and integrated into further assessment. A schematic overview of the included studies, including population characteristics, dual-task characteristics, and key findings, is provided in Table 1.

### Critical appraisal

The methodological quality of the eight included studies was evaluated using a modified 18-item Downs and Black (1998) checklist, yielding total scores ranging from 9 to 16 (Downs and Black 1998). Antonellis et al. (2025) achieved the highest quality rating (16/18), whereas Cossette et al. (2014) and Damiano et al. (2016) received the lowest scores (9/18). Generally, the included literature demonstrated sufficient reporting quality, with the majority of studies satisfying criteria regarding the clarity and completeness of data. However, common limitations were identified in the external validity and power sub-scales, indicating widespread challenges with generalizability and sample size justification across the dataset. An overview of the methodological quality evaluation and a description of the included items is provided in Table 1.

**Table 1:** Methodological quality assessment of included studies

Author(s)	Year	R1	R2	R3	R4	R5	R6	R7	R8
Antonellis et al.	(2025)	Yes	Yes	Yes	Yes	No	Yes	Yes	Yes
Joseph et al.	(2021)	Yes	Yes	Yes	Yes	Yes	No	Yes	Yes
Martini et al.	(2021)	Yes	Yes	Yes	Yes	Yes	Yes	Yes	Yes
Bryk et al.	(2023)	Yes	Yes	Yes	Yes	Yes	Yes	Yes	Yes
Bryk et al.	(2024)	Yes	Yes	Yes	Yes	Yes	Yes	Yes	Yes
Geurts et al.	(1996)	Yes	Yes	Yes	Yes	No	Yes	Yes	No
Cossette et al.	(2014)	No	No	No	Yes	No	No	No	Yes
Damiano et al.	(2016)	Yes	Yes	No	No	No	Yes	Yes	Yes

EV1	EV2	EV3	EV4	EV5	EV6	IV1	IV2	IV3	*P*	Sum score
Yes	Yes	No	Yes	Yes	Yes	Yes	Yes	Yes	Yes	16
no	No	No	Yes	Yes	Yes	Yes	Yes	Yes	Yes	14
no	No	No	Yes	Yes	Yes	No	Yes	Yes	Yes	14
no	No	No	Yes	Yes	Yes	Yes	Yes	No	No	13
no	No	No	No	Yes	Yes	Yes	Yes	Yes	No	13
no	No	No	Yes	Yes	Yes	No	Yes	Yes	No	11
Yes	Yes	No	Yes	Yes	Yes	Yes	Yes	No	No	9
no	No	No	Yes	Yes	Yes	No	Yes	No	No	9

Studies were assessed using a modified Downs and Black checklist consisting of 18 items across four sub-scales: Reporting (R1–R8; assessing clarity and completeness of data); External Validity (EV1–EV6; assessing generalizability to the wider population); Internal Validity (IV1–IV3; assessing measurement bias and adjustment for confounding); and Power (P1; assessing the sufficiency of the sample size)

### Populations included

**Fig. 2:**
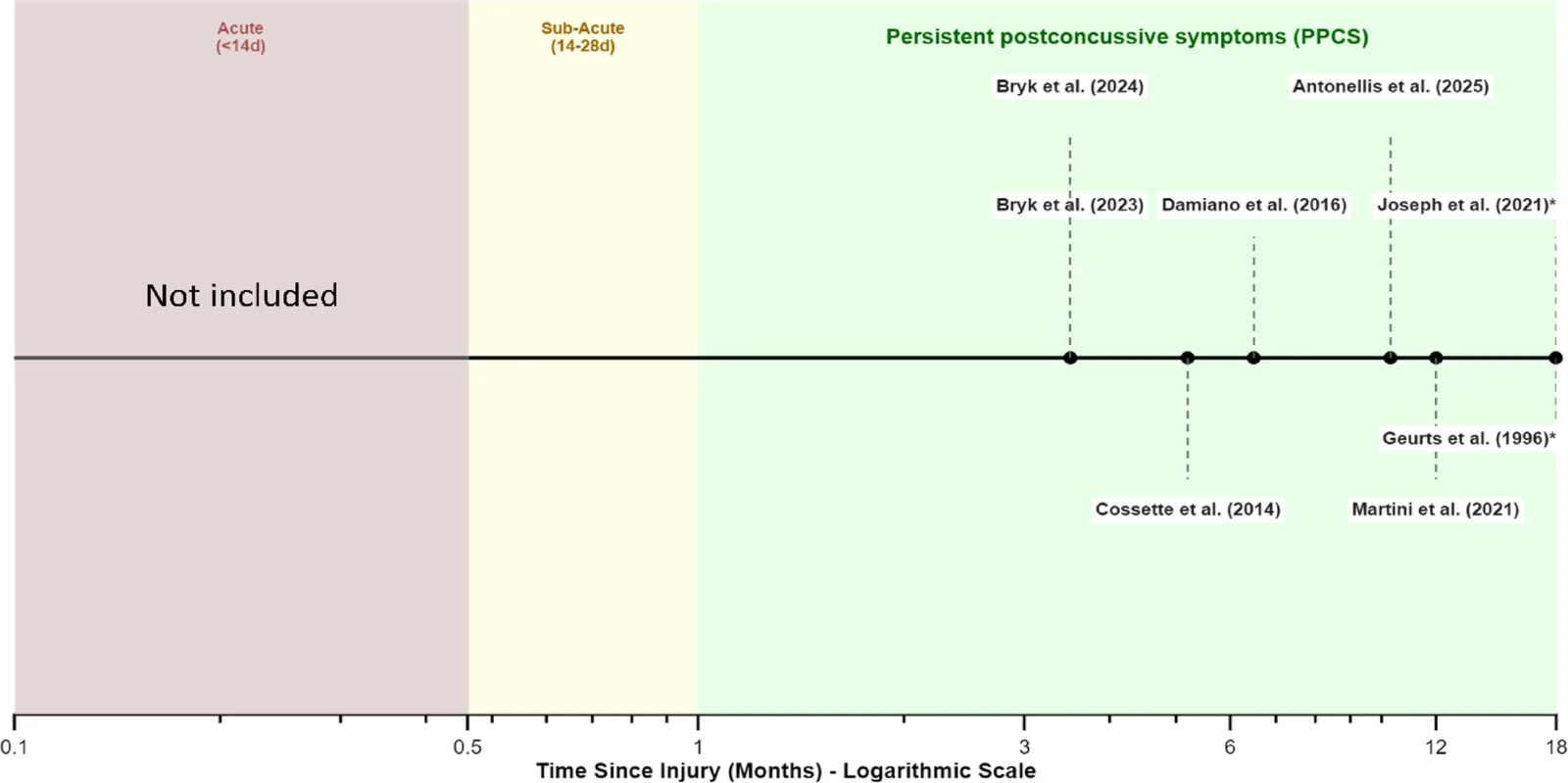
Timeline of included studies based on time since concussion. The horizontal axis displays the time since injury in months on a logarithmic scale. Injury phases are categorized into acute (< 14 days, red shading), sub-acute (14–28 days, yellow shading), and persistent postconcussive symptoms (PPCS, green shading)

In total, the eight studies included in the present review elaborated a cohort of *N* = 197 patients (37.3 + 12.9 years; 131 females). The mean time between injury and dual-task assessment ranged between three months (Bryk et al. 2023, 2024) and 29 months (Geurts et al. 1996). Notably, systematic evaluation of the literature identified not a single study within the subacute period (14 days to one month) after concussion that investigated DTC alongside symptom burden (see Fig. 2).

The mean ages ranged between 20 and 46 years. For the identification of PPCS symptoms, different symptom inventories were used, including the Neurobehavioral Symptom Inventory (*n* = 3 (Soble et al. 2014)) and Rivermead Post-Concussion Symptoms questionnaire (*n* = 3 (King et al. 1995)) to address overall symptom burden. Some studies specified the NSI subscales to further specify the symptom burden in the cognitive, vestibular, somatosensory, or anxiety domain. The Beck Anxiety Inventory (BAI; (Eck et al. 2025)), the Posttraumatic Stress Disorder Checklist-Civilian Version (PCLC; (Conybeare et al. 2012)), and the Beck Depression Inventory (BDI; (Aalto et al. 2012)) were each used once and indicated inventories to assess symptom burden in the anxiety symptom cluster. The Dizziness Handicap Inventory (DHI; (Duong Dinh et al. 2022)) was used once and indicated an inventory assessing the vestibular symptom cluster.

### Dual-task protocols utilized

On the motor side, six out of the eight studies assessed gait as primary motor function (Antonellis et al. 2025; Bryk et al. 2023, 2024; Cossette et al. 2014; Joseph et al. 2021; Martini et al. 2021). While four of the six studies assessed normal gait, two studies included gait variations such as obstacle gait and stepdown gait (Cossette et al. 2014) or gait initiation (Bryk et al. 2024). All six studies assessed gait from overground walking instead of motorized treadmill walking. While five studies conducted the gait assessment in laboratory or controlled corridors, one study (Antonellis et al. 2025) assessed gait in public hospital hallways. All studies quantified gait speed as the primary outcome, while single studies also reported on more specific gait parameters such as gait variability, gait rhythm, step length, step width, and turning. Two studies assessed static balance through stance tasks (Damiano et al. 2016; Geurts et al. 1996). One study used the NEUROCOM system (Damiano et al. 2016) to quantify balance, while one study used a conventional force plate (Geurts et al. 1996). While Damiano et al. 2016 quantified balance by means of area of sway, Geurts et al. 1996 quantified balance performance by means of sway velocity during weight shifts.

On the cognitive side, tasks were categorized into working memory tasks, inhibition tasks, and verbal fluency tasks. Six studies utilized working memory tasks to induce cognitive load, and all studies applied an arithmetic counting backwards task (Antonellis et al. 2025; Cossette et al. 2014; Damiano et al. 2016; Geurts et al. 1996; Joseph et al. 2021). Bryk et al. (2024) conducted a series of working memory tasks, including spelling backwards, listing months backwards, and the aforementioned arithmetic tasks. Joseph et al. 2021 also applied an n-back paradigm to induce working memory load. Further, four studies used inhibition tasks by means of either visual (*n* = 2 (Bryk et al. 2023; Cossette et al. 2014)) or auditory (*n* = 2 (Bryk et al. 2024; Martini et al. 2021)) versions of the Stroop task. Further, thre studies used verbal fluency tasks (Bryk et al. 2023; Cossette et al. 2014; Damiano et al. 2016) to induce cognitive load. For all studies, performance was quantified by means of accuracy and errors, while none of the included studies using computerized stimulus-based paradigms reported reaction or response times, respectively. After contacting authors, three studies (Bryk et al. 2023, 2024; Damiano et al. 2016) provided reaction time data for the Stroop task paradigm. An overview of dual-tasks utilized is provided in Table 2.

**Table 2:** Overview of included studies characteristics with a specific focus on the applied dual task paradigms

Authors	Dual task						
	Motor		DTCanalysis?	Cognitive			DTCanalysis?
	Task(s)	Outcome(s)		Task(s)	Accuracyoutcome(s)	Timeoutcome(s)	
(Antonellis et al. 2025)	Gait Hospital hallway	Gait speed Gait variability Gait rhythm Turning	Y	Working memory: Arithmetic [A]	Not available	Not available	N
(Bryk et al. 2023)	Gait Overground	Gait speed Step length Step width	Y	Inhibition: visual stroop [VS] Verbal fluency: word recall [WR] Working memory: spelling backwards, listing months backwards, arithmetic tasks [WM]	Accuracy (VS, WM) Word count (WR)	Response times (VS, after request)	Y
(Bryk et al. 2023)	Gait Overground	Gait initiiation	Y	Inhibition: Auditory Stroop [AS]	Accuracy (AS)	Response times (AS, after request)	Y
(Cossette et al. 2014)	Gait (a) Obstacle gait (b) Stepdown Gait overground	Gait speed	Y	Inhibition: visual stroop [VS] Verbal fluency. word recall [WR] Working memory: arithmetic [A]	Not available	Not available	N
(Damiano et al. 2016)	Static balance Neurocom	Composit score	Y	Verba fluency: word recall [WR] Working memory: arithmetic [A]	Word count (WR) Number count (A)	Not available	N
(Geurts et al. 1996)	Static balance Weight shift Forceplate	Sway velocity Weight shifts	Y	Working memory: arithmetic [A]	Errors (A)	Not available	Y
(Joseph et al. 2021)	Gait Overground	Gait speed	Y	Working memory: arithmetic [A]	Number count (A)	Not available	Y
(Martini et al. 2021)	Gait Overground	Gait speed, Gait variability Gait rhythm	Y	Inhibition: auditory stroop [AS]	Accuracy (AS)	Response times (AS, after request)	Y

### Findings on CMI in PPCS

Seven out of eight reviewed studies indicate that PPCS cohorts exhibit statistically significant impairments during dual-task performance compared to healthy controls. Across diverse cohorts ranging from young adults (Cossette et al. 2014) to middle-aged populations (Joseph et al. 2021), the most prominent finding was a marked increase in dual-task cost during gait. Specifically, PPCS cohorts demonstrate altered motor strategies characterized by increased gait variability, shorter step lengths, and slower walking speeds when simultaneously performing a cognitive task (Antonellis et al. 2025; Bryk et al. 2023). More specific gait parameters related to turning and rhythmic movement during walking were also notably affected (Martini et al. 2021). While all studies analyzed the motor domain for dual-task cost analysis, only four out of eight studies considered cognitive domain performance for dual-task cost analysis. Only one study considered both changes in motor performance and cognitive performance to evaluate task priorization strategies and observed a cognitive priorization strategy, where patients maintained cognitive accuracy at the expense of motor stability (Bryk et al. 2023). While a single study reported no significant change in static postural control (Geurts et al. 1996), six studies suggested that dynamic tasks, such as walking or navigating obstacles, are impaired in PPCS cohorts. An overview of the results of the included studies is provided in Table 3.

**Table 3:** Overview of characteristics of the included studies with a specific focus on patient descriptives, symptom inventories used and the main findings

Authors	Type	Findings					
		Total n Patients n Female n	Age (patients)	Time since injury	Symptom inventory	Main findings group x dual-task interaction	
(Antonellis et al. 2025)	C	107 Participants 52 Patients 42 females	31.9 ± 9.4	313.1 ± 261.4 D	Overall: NSI	MOT:	↑ Increased DT Turn Duration for PPCS (*p*=.004; d=0.579) ↓ Reduced DT Turn Velocity for PPCS (*p*=.006; d=0.267) ↑ Increased DT single support time for PPCS (*p*=.002; d=0.610)
						COG:	*Not analyzed*
(Bryk et al. 2023)	C	38 Participants 15 Patients 11 Females	43.9 ± 11.7	> 3 M	Overall: RPSQ-13	MOT:	↓ Reduced DT gait speed for PPCS (*p*=.009; d=0.92) ↓ Reduced DT step length for PPCS (*p*=.023; d=0.76)
						COG:	↓ Reduced DT Working Memory Accuracy for PPCS (*p*=.008; d=0.96)
(Bryk et al. 2024)	C	38 Participants 15 Patients 11 Females	43.9 ± 11.7	> 3 M	Overall: RPSQ-13	MOT:	↑ Increased DT antero-posterior sway displacement for PPCS (*p*=.016, d = 0.874) ↑ Reduced DT medio-lateral sway velocity for PPCS (*p*=.041, d = 0.866)
						COG:	→ no difference in DTC for the auditory Stroop task accuracy (*p*=.321, d = 0.304)
(Cossette et al. 2014)	C	14 Participants 7 Patients 6 Females	20.0 ± 1.6	158.4 ± 130.3 D	Overall: RPSQ-3	MOT:	↓ Reduced DT gait speed for PPCS across gait tasks (p = < 0.001, d=0.89)
						COG:	*Not analyzed*
(Damiano et al. 2016)	C	24 Participants 12 Patients 5 Females	31.3 ± 9.4	> 6 M	Overall: PSQI Anxiety: BAI Anxiety: PCLC	MOT:	↓ Reduced DT standing balance for PPCS (*p*=.047, d = not reported)
						COG:	*Not reported*
(Geurts et al. 1996)	C	40 Participants 20 Patients 9 Females	36.2 ± 10.7	28.6 ± 25.7 M	Overall: GCS	MOT:	↑ Increased DT lateral sway during static balance for PPCS (*p*<.05; d = *nr*) ↑ Increased DT lateral sway during dynamic balance for PPCS (*p*<.01; d = *nr*)
						COG:	→ No interaction effect observed for counting errorrs (*p*=.093 d = *nr*)
(Joseph et al. 2021)	C	52 Participants 26 Patients 13 Females	46.6 ± 16.6	584.5 ± 304.3 D	Overall: NSI Vestibular: NSI Somatosensory: NSI Cognitive: NSI Anxiety: NSI Vestibular: DHI Anxiety: BDI	MOT:	→ No enhanced DTC during static balance for PPCS ↓ Reduced DT gait speed for PPCS (*p*=.036; d=0.6)
						COG:	↓ Reduced DT cognitive performance for PPCS (*p*=.03; d=0.64)
(Martini et al. 2021)	C	122 Participants 65 Patients 45 Females	39.6 ± 11.7	1.0 ± 13.0 Y	Overall: NSI Cognitive: NSI Anxiety: NSI	MOT:	↓ Slower pace, turning, and rhythm during DT for PPCS (*p*<.001; d = 0.64 to 0.87)
						COG:	↓ Reduced Stroop Accuracy during DT for PPCS (*p*=.021; d = 0.48)

Neurobehavioral Symptom Inventory (NSI), Rivermead Post-Concussion Symptoms Long (RPSQ-13), Rivermead Post-Concussion Symptoms Short (RPSQ-3), Pittsburgh Sleep Quality Index (PSQI), Beck Anxiety Inventory (BAI), PTSD Checklist-Civilian Version (PCLC), Glasgow Coma Scale (GCS), Dizziness Handicap Inventory (DHI), Beck Depression Inventory (BDI); SR = Self-Reported Symptoms

### Concept mapping

**Fig. 3:**
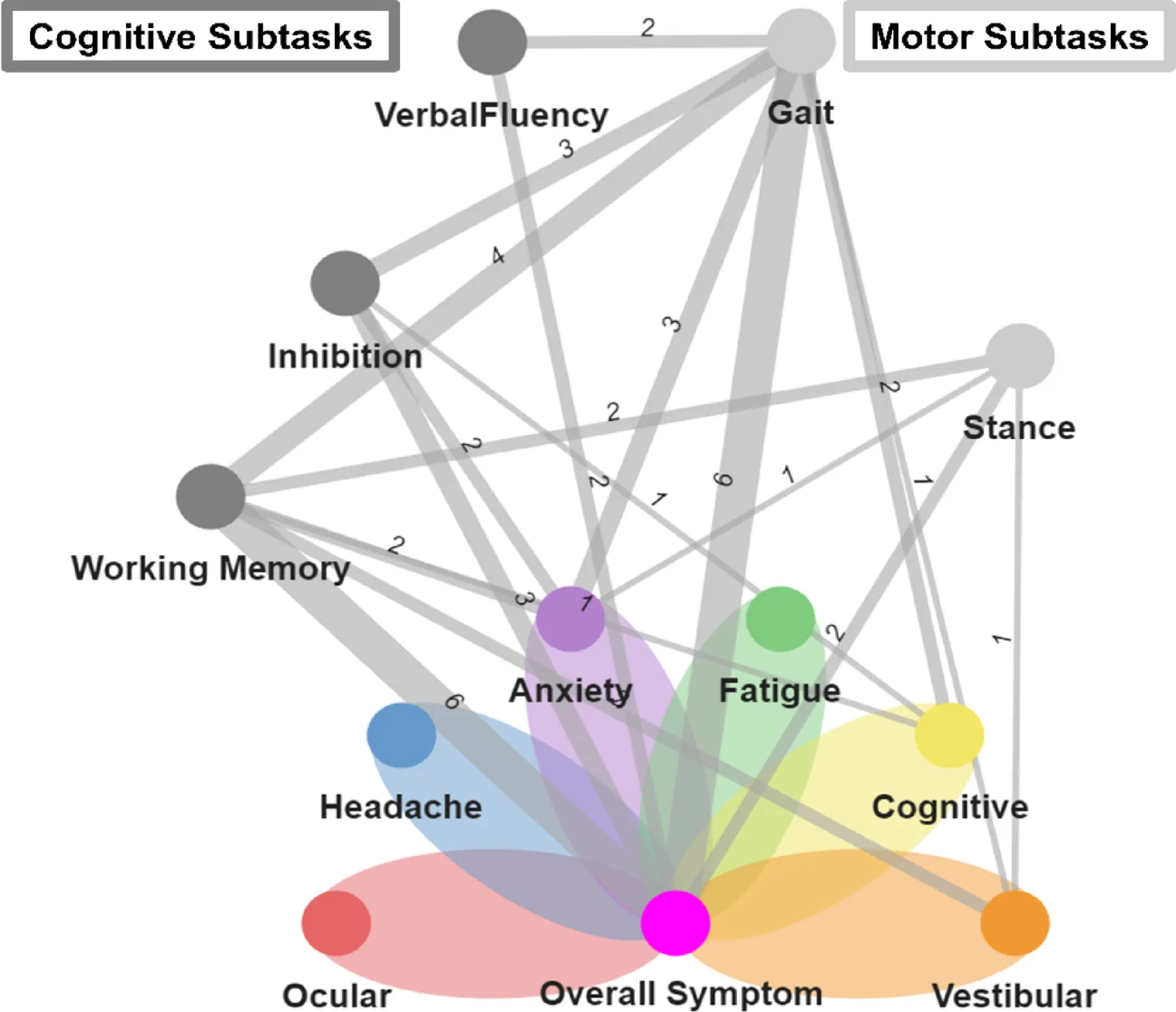
Concept map illustrating co-occurrences between motor subtasks, cognitive subtasks, and symptom domains in the current literature (*n* = 8 studies) on individuals with persistent postconcussive symptoms. Connecting lines indicate the presence of an association, with numerical labels and varying line thicknesses representing the frequency of co-occurrences

Based on the extracted data of the eight included studies, a concept map was drafted to visualize the co-occurrence of the a priori defined PPCS symptom domains and the post-hoc evaluated cognitive tasks (light grey nodes) and motor tasks (dark grey nodes). The concept map is presented in Fig. 3. It is crucial to note that the edges in this map strictly represent the co-investigation of these variables within the same experimental protocol, not biological or clinical relationships. A dominant finding is the central role of “Overall Symptom Burden,” acting as a prominent hub connecting executive functions of Inhibition (Stroop) and Working Memory (arithmetic, word recall) with motor tasks. On the motor outcome side, “gait” demonstrates stronger nodes than “stance,” suggesting that it is the primary physical metric studied in PPCS. While the symptom concepts “Vestibular” and “Anxiety”, and “Cognitive” show edges to gait, symptoms like “Headache”, “Fatigue”, “Ocular,” appear without edges to either cognitive or motor tasks. The mapping further reveals that both working memory and inhibitions share edges with motor outputs. Overall, the analysis indicates a heavy research focus on the interplay between broad symptom burden, working memory, and dynamic motor control.

## Discussion

The overarching aim of this scoping review was to summarize the existing literature on dual-task applications in patients with symptoms lasting beyond the acute phase after concussion, both including relevantly affected patients in the subacute phase at risk of persisting impairments and patients with already persisting postconcussive symptoms (PPCS). Here, we identified a sample of eight studies, all of which assessed patients with persisting symptoms of more than one month (i.e., PPCS). Of these, seven studies support our initial assumption that CMI is elevated in PPCS cohorts. Accordingly, the parallel assessment of motor and cognitive functions appears a promising approach to address symptom burden in PPCS.

Our secondary aim was to identify which motor and cognitive subtasks are most frequently investigated in PPCS dual-task research. Here, we observed that the majority of studies used gait as a motor task, while the domains of working memory, response inhibition and verbal fluency were similarly distributed among the cognitive tasks. Therefore, the combination of gait with executive functions tasks like inhibition or working memory seems a feasible combination to expose functional deficits in individuals with PPCS. Notably, a shortcoming in reporting time-domain outcomes for the cognitive subtasks unravels further potential for future studies.

Finally, we aimed to map associations between the concussion symptomdomains identified by Harmon et al. (2019) with the identified motor and cognitive subtasks. Here, we observed an underrepresentation of specific concussion symptoms such as ocular dysfunction, fatigue, and headache, while more generically assessed symptoms were most frequently addressed in the current literature. Therefore, our concept mapping identifies a current shortcoming with respect to specific concussion subdomains. Further, the strong associations between working memory, inhibition, and gait revealed important implications for future dual-task experiments with PPCS cohorts.

### Key finding I: deficits in cognitive-motor integration in PPCS

The main finding of the present synthesis of the literature was that seven out of eight studies observed increased difficulties in performing a motor and a cognitive task in parallel for PPCS cohorts. These findings highlight the potential of specific dual-task investigations in PPCS cohorts to serve as sensitive research metrics that expose underlying vulnerabilities in neural resource allocation. While these tasks effectively expose cognitive-motor integration challenges, future studies must aim to establish the specific clinical thresholds at which these statistical differences result in meaningful functional problems for the individual. The most recent included studies by Bryk et al. (2023) and Antonellis et al. (2025) revealed that these differences between PPCS and controls were only present under dual-task, but not ST conditions. These findings suggest that competition for limited neural resources represents a key deficit in individuals with PPCS compared to healthy controls, thereby interfering with cognitive-motor integration (Howell et al. 2018). Accordingly, integrating dual-task assessments into rehabilitation monitoring may complement existing subjective measures, such as the Rivermead Post-Concussion Symptoms Questionnaire (King et al. 1995).

Although PPCS cohorts require medical and rehabilitative treatment due to lasting postconcussive symptoms affecting everyday life (Machamer et al. 2022), we only identified a small number of eight studies that applied dual-task in this group of patients. While some more studies would have met our inclusion criteria regarding time from injury and dual-task paradigm, many studies did not track symptoms based on an evaluated concussion symptom inventory (e.g., (Fino et al. 2018)). Therefore, further research on the concussion subgroup of PPCS with specific regard to specified symptom burden is lacking in the current dual-task literature, since the present review is the first one at all to focus explicitly on individuals with PPCS more than 14 days after injury. In consequence, current dual-task practices on PPCS cohorts are likely derived from research on acute concussion cohorts. These conclusions may induce pitfalls related to the different underlying neural mechanisms resulting in increased CMI for acute concussions, subacute symptoms, and PPCS, respectively. While increases in CMI in the acute concussion phase may result from neurometabolic deteriorations which dampen neural processing speed and integration (Giza and Hovda 2001), these neurometabolic deteriorations are presumably recovered in PPCS, with network reorganization and biopsychological factors contributing to symptom persistence (Willer and Leddy 2006). Consequently, the development and characterization of specific dual-task protocols for PPCS cohorts is important, and the included subtasks should target relevant symptom domains, such as fatigue and anxiety.

### Key finding II: combining gait and executive functions to challenge PPCS cases

Our analysis revealed that gait is the dominant motor task investigated in dual-task experiments in PPCS. All studies investigating gait found a slowing of gait in combination with a parallel cognitive task. The most frequently reported phenomena were reductions in gait velocity and step length compared to controls under dual-task conditions (Antonellis et al. 2025; Bryk et al. 2023; Martini et al. 2021). Further, more specific parameters such as gait initiation (Bryk et al. 2024), obstacle gait (Cossette et al. 2014), or gait turns (Martini et al. 2021) significantly slowed down in PPCS under dual-task conditions. In contrast, only two studies examined standing balance (Damiano et al. 2016; Geurts et al. 1996), and only Damiano et al. (2016) observed increased postural sway in the PPCS cohort under dual-task conditions. According to the higher relevance of walking for complex daily activities, we recommend gait as a preferable motor domain to investigate dual-task costs in PPCS cohorts.

However, there was a high variability of approaches used to assess gait, with resulting methodological heterogeneity across studies (Mitchell and Cronin 2023). Of the studies described here, some used gold-standard motion capturing (Cossette et al. 2014) or highly sensitive force plate measures (Bryk et al. 2024), while others applied wearable sensor technologies (Antonellis et al. 2025). While the latter offer increased ecological validity, they are accompanied by increased measurement errors (Yin et al. 2025). Similar issues are evident in the choice of specific gait outcome measures. In the current literature, most studies investigate established metrics such as gait speed or variability, which can be assessed reliably across technologies (Rudisch et al. 2021; Yin et al. 2025). However, more specific but ecologically more valid measures such as gait turns (Antonellis et al. 2025) and initiations (Bryk et al. 2024) require more complex processing pipelines, which reduce the reproducibility of findings. These heterogeneities in technologies used and outcomes assessed require a careful trade-off between external and ecological validity to utilize dual-task costs as an objective marker of functional recovery in PPCS. Therefore, we recommend that future studies consider the combination of both established gait features revealed from overground walking with more explorative approaches such as gait initiations and gait quality during turns to investigate CMI in PPCS.

In contrast to the motor subtasks, the current evidence revealed a more heterogeneous landscape with regard to the added cognitive subtasks (Fig. 3). The most frequently applied cognitive tasks were serial subtraction tasks (*n* = 5), followed by visual or auditory Stroop tasks (*n* = 4) and verbal fluency tasks (*n* = 3). When applied to the concept of executive functions by Diamond (2013), these task paradigms majorly reflect the core executive functions of working memory (keeping information in mind; serial subtraction) and inhibition (selective attention; Stroop task) (Diamond 2013). Additionally, verbal fluency tasks were frequently employed, serving as broader, frontally mediated EF measures that require strategic retrieval and cognitive control (Baldo et al. 2006). Otherwise, cognitive flexibility, the third domain of executive function, is currently poorly reflected in dual-task experiments in PPCS. These findings are in line with a recent taxonomy paper on dual-task research in older adults, where arithmetic and verbal fluency tasks were the most frequently assessed tasks (Wollesen et al. 2019), potentially reflecting a convenience choice, since both verbal tasks do not require extensive programming or dedicated software licenses (Wollesen et al. 2019). Overall, both computerized and verbal tasks relying on core EFs seem to induce increased CMI in PPCS cohorts.

Notably, only four out of eight studies considered cognitive domain performance for dual-task cost analysis. Thus, task priorization analyses as performed by Bryk et al. (2023) remain rare, but could provide distinct insights into compensational strategies depending on symptom burden (Bryk et al. 2023). Therefore, it might be possible that the lack of reporting on cognitive dual tasks may mask dual-task costs and potential cognitive prioritization strategies in PPCS. Further, an analysis of the investigated outcomes revealed that all articles based their calculations of cognitive dual-task costs on accuracies and error counts, but none of the studies reported response times. Response times describe the time from stimulus presentation to motor response and reflect processing speed capacities of theindividual (Woods et al. 2015). While such metrics are typically provided for computerized tasks, they are not assessed for arithmetic or verbal fluency tasks. Nevertheless, combining error rates and response times allows for the calculation of inverse efficiency scores to determine the speed-accuracy tradeoffs of patients under dual-task conditions (Fitts 1966). After requesting response times from the authors with computerized tasks, we obtained data from three studies using the Stroop task (Bryk et al. 2023; Martini et al. 2021). Response time data revealed delayed responses for PPCS cohorts in all three studies, demonstrating that the overall efficiency of the individuals was reduced in PPCS cohorts. Since the trade of speed for accuracy has been previously described as a compensatory strategy in concussed athletes (Battistone et al. 2008), combining response time and accuracy may reveal more holistic insights into cognitive inefficiency in PPCS patients. Therefore, we recommend that future dual-task studies prefer computerized tasks and include both accuracy and time metrics. Such approaches would also enhance the objectivity of the assessment of dual-task costs.

To advance beyond behavioral metrics and explore the neurophysiological mechanisms of CMI in PPCS, mobile neurophysiological methods represent a highly valuable methodological approach for future dual-task research (Gramann et al. 2014). By synchronizing high-density mobile electroencephalography (EEG) with full-body kinematics and computerized cognitive tasks, mobile neurophysiological methods allow researchers to observe how the brain reallocates resources during unconstrained movement. High channel counts are particularly advantageous in these dynamic settings, as they enable superior spatial filtering and independent component analysis to effectively reject motion and muscle artifacts. Recent studies quantified attentional reallocation during real-world walking (Ladouce et al. 2019; Reiser et al. 2021) and serve as a potential orientation for future PPCS dual-task research. Furthermore, functional near-infrared spectroscopy (fNIRS) represents a highly robust complementary modality that is less susceptible to motion artifacts (Helmich et al. 2020). Ultimately, combining both high-density EEG and fNIRS may represent a promising approach, combining EEG for precise temporal resolution alongside fNIRS for the spatial resolution of cortical oxygenation. Together, these modalities could reveal insights into the specific neural bottlenecks driving CMI, offering direct, objective physiological markers of functional recovery that behavioral tests alone cannot provide. Although such multimodal methodologies are currently highly experimental and confined to specialized research facilities, these pivotal investigations are essential for the development of clinical diagnostics.

Taken together, the combination of a motor and a cognitive subtask, while assessing performance of each, is a promising approach to track functional recovery of PPCS cohorts objectively across six studies. While gait displays a crucial motor function and allows individuals to participate in dynamic environments, core executive functions are also crucial for everyday life and essential to produce and regulate complex goal-directed behavior (McAlister and Schmitter-Edgecombe 2016). Therefore, changes in gait patterns, reductions in task accuracy, or reduced response times under dual-task conditions reflect a dysfunctional integration of basal motor and cognitive processes in PPCS, which reduce quality of life (Doroszkiewicz et al. 2021). Such functional deficits may contribute to the manifestation of anxiety, depression, and fatigue in the target group and result in a mutual reinforcement of biological and psychological symptoms leading to chronification of deficits (Doroszkiewicz et al. 2021). This mutual interaction is also a key mechanism differentiating acute concussions from PPCS.

While simple isolated tests may expose deficits based on neurometabolic irritations in acute concussions, network reorganization in the subacute period compensates for impairments in simple tasks (Churchill et al. 2021). However, the reinforcement between sensorimotor and psychological symptoms clusters according to Harmon et al. (2019) may result in a performance-detrimental bottleneck (Ruthruff et al. 2001) during complex cognitive-motor tests and further advocates for the implementation of dual-tasks into PPCS monitoring. In this context, task difficulty and complexity must be carefully considered when designing these assessments. Difficulty reflects the interplay between the complexity of a specific task demand and the individual’s capacities (Chen et al. 2023). The latter can be moderated by acute stressors such as postconcussive symptom severity. For diagnostic testing, the difficulty threshold should be high enough to expose underlying deficits; for example, simple stance tasks may underchallenge individuals with predominantly cognitive symptoms, failing to induce measurable CMI. Conversely, in therapeutic settings, task difficulty requires careful, individualized modulation. Here, the present review supports the combination of gait and inhibitory control tasks such as the Stroop paradigm, providing objective insights into disturbances of locomotor control and cognitive efficiency.

### Key finding III: lack of symptom-specificity in PPCS dual-task research

Finally, we mapped associations between the core concussion symptom clusters defined by Harmon et al. (2019) and dual-task subtasks. Interestingly, the majority of studies provided summary scores obtained from the Neurobehavioral Symptom Inventory (NSI) or the Rivermead Post Concussion tool, while few studies addressed specific symptom domains such as anxiety (Becks-Anxiety-Index; BAI), depression (Becks-Depression-Index; BDI), or sleep (Pittsburgh-Sleep-Index; PSI). Further, vestibular and cognitive dysfunction symptom burden was solely reported using the subscales of the NSI. Moreover, specific ratings on core symptom clusters such as fatigue, headache, or ocular dysfunction (Harmon et al. 2019) were not investigated specifically. Therefore, our mapping analysis revealed a shortcoming of specific symptom score reporting. Considering that PPCS distinguishes itself from acute concussion due to its biopsychological chronification with important contributions from anxiety and fatigue (Doroszkiewicz et al. 2021), these essential symptoms appear underrepresented. Therefore, we recommend that future studies on PPCS emphasize the assessment of psychological symptoms for a better understanding of the mutual interactions between psychological symptoms and dual-task costs.

Besides the lack of specific symptom scores, our concept map revealed strong associations between general symptom scores, gait, and arithmetic tasks or inhibition tasks. Further, anxiety displayed an important hub in the concept map and shared strong connections with gait and inhibitory control. These findings emphasize the role of anxiety as a key driver in PPCS, which arises and mediates between posttraumatic stress symptoms, cognitive coping strategies, and trauma sequelae (Donlon and Jones 2015). In particular, athletes with increased pre-injury anxiety seem to need more time to recover from concussion symptoms and return to pre-injury performance level (Huang et al. 2024). Since anxiety itself is expected to bind attentional resources through internal conflict processes, increased anxiety is expected to reduce working memory (Moran 2016) and inhibitory control (Wood et al. 2001). Therefore, anxiety and depression should be considered as key moderators for increased dual-task costs in PPCS cohorts and specifically addressed in dual-task research. For instance, researchers should individually capture anxiety burden such as the Generalized Anxiety Disorder Index-7 (Roberts et al. 2024) as a potential moderator for dual-task costs. Further, our concept map revealed cognitive and vestibular dysfunctions as smaller nodes. For instance, cognitive dysfunction was linked to inhibition and gait, while vestibular dysfunction was linked to stance and working memory and gait and inhibition, respectively (see Fig. 3). These specific interactions between more isolated symptom clusters and dual-task subtasks underscore the need for a careful design of dual-task assessments in the heterogeneous cohort of PPCS, with the goal of developing a universal approach to monitor biological recovery. While simple stance tasks on a stable surface could be particularly promising in subjects with vestibular symptoms, they may underchallenge individuals with rather psychological or cognitive symptoms (Gebel et al. 2020). On the other hand, simple cognitive tasks may challenge patients with cognitive symptoms, but may not induce CMI in cognitive functional subgroups (Wollesen et al.^2019^). Therefore, combining gait with core executive function tasks might be the most promising approach to induce dual-task costs across PPCS symptom subgroups with special regard to individual task loadings.

### Limitations

While this scoping review provides an important first synthesis of dual-task applications with a focus on PPCS, several limitations must be acknowledged. First, the small number of identified studies (*n* = 8) limits the generalizability of the findings. Accordingly, we conducted a scoping review to explore the field and highlight underrepresented areas of research. At the same time, there was considerable heterogeneity in participant demographics, ranging from athletes to veterans across various age groups. This diversity complicates the establishment of universal normative values for dual-task performance in PPCS.

Further, we observed significant variance in the technologies used (e.g., gold-standard motion capture vs. wearable sensors) and the specific subtasks employed. This lack of standardization makes direct comparisons of effect sizes across studies difficult and highlights the need for a consensus on dual-task protocols (Olsen et al. 2026). The concept mapping aims to guide decision-making for upcoming dual-task studies on the target cohort. While the symptom flower of Harmon et al. (2019) addresses recurring clinical profiles of PPCS, it barely addresses more specific target symptoms such as exercise tolerance or sensorimotor integration. Therefore, the present concept map does not claim to cover all potential symptoms and may neglect specific outcomes such as exercise tolerance (Leddy et al. 2018). Further, it must be noted that the majority of articles reported overall symptom burden based on the NSI or Rivermead questionnaire. Therefore, more specific data on symptom clusters and their impact on dual-task costs might be available in clinical databases, but are not yet addressed in the published evidence. Additionally, the lack of standardized reporting for cognitive outcomes, specifically the omission of response times in many studies, limits our understanding of specific compensational strategies such as speed-accuracy trade-offs. Without these metrics, the full extent of reduced processing efficiency in PPCS is likely to be underestimated. Finally, all included studies were cross-sectional. Consequently, the current evidence does not allow for evaluating how dual-task performance and symptom burden change longitudinally over the course of recovery. Therefore, there is a lack of evidence investigating whether dual-task costs can serve as a sensitive marker for the transition from the symptomatic to the asymptomatic state. It is important to note that longitudinal dual-task data *do* exist in the broader concussion literature (Catena et al. 2009; Howell et al. 2015). However, these studies were excluded from our conceptual mapping because they relied on unsystematic self-reporting rather than validated symptom inventories. While our stringent inclusion criteria were necessary to map specific clinical clusters reliably, the exclusion of these longitudinal studies means our current sample lacks evidence regarding how dual-task costs develop over time, or whether they can serve as a sensitive predictive marker for the transition from a symptomatic PPCS state to full recovery.

## Conclusions

This scoping review highlights that dual-task assessments, particularly the combination of walking and executive function tasks (such as working memory and response inhibition), are a highly sensitive and valuable approach to unmask subtle CMI deficits in PPCS patients. Unlike acute concussions, where CMI deficits stem from transient neurometabolic dysfunction, PPCS is characterized by complex, mutually reinforcing biopsychological mechanisms and functional (e.g., compensatory or aberrant) network adaptations. However, our review reveals critical gaps in the current literature: specific symptom clusters (e.g., fatigue, ocular dysfunction) are rarely systematically tracked, and precise temporal outcomes like cognitive response times are frequently omitted. To address these shortcomings, future studies must standardize dual-task methodologies by utilizing computerized cognitive tasks to capture speed-accuracy trade-offs accurately. Furthermore, to map the neural correlates underlying these behavioral deficits and bypass compensatory strategies, we strongly advocate for the integration of neurophysiological methods such as EEG and fNIRS paradigms into PPCS research. Information on the efficiency and localization of neural processes underlying PPCS symptoms can support the development of targeted intervention and rehabilitation strategies. Further, such information could provide novel insights into the functional reorganization of the CNS in the transition from acute to subacute and PPCS eventually. Ultimately, refining dual-task protocols will provide researchers and clinicians with sensitive, ecologically valid tools to track cognitive-motor integration. While further research is needed to translate these statistical differences into everyday functional thresholds, assessing these domains in parallel represents a crucial step toward tailoring comprehensive rehabilitation strategies for individuals with PPCS.

## Supplementary Information

Below is the link to the electronic supplementary material.

Supplementary Material 1

## Data Availability

No datasets were generated or analysed during the current study.
