# Supplementary material for: Dual-task testing in adults with persistent postconcussive symptoms: a scoping review with conceptual mapping approach
Source: Exp Brain Res. 2026 Sep 6;244(10):195. doi: 10.1007/s00221-026-07392-w (PMC13547169; doi:10.1007/s00221-026-07392-w)
Supplement: Supplementary file 1 [file 221_2026_7392_MOESM1_ESM.docx]

| POPULATION    INTERVENTION    OUTCOME    SYMPTOMS    STUDY TYPE | ("post-concussion" OR “postconcussion” OR concussive OR "mild traumatic brain injury" OR "mTBI" OR concussion OR "head injury" OR "vestibular dysfunction" OR dizziness OR drowsiness OR sadness OR “pressure in head” OR sensitivity OR “neck pain” OR tenderness  OR “blurred vision*” OR migraine* OR headache OR migrainous OR “impaired vision” OR “ocular dysfunction” OR "cognitive fatigue" OR “mental fatigue” OR depression OR fear OR anxiety OR irritability OR fatigue OR insomnia OR sleep OR somnolence OR “confusion” OR “energy deficit”) AND (“adult*” OR “adults” OR “middle-aged” OR “middle-aged”)  AND  "dual-task" OR "dual task" OR "dual-tasking" OR "concurrent task" OR “coupled task"OR balance OR "divided attention" OR gait OR mobility OR “postural control” OR "simultaneous task" OR "motor-cognitive" OR "cognitive-motor" OR "cognitive task" OR "motor task" OR "dual task paradigm" OR "secondary task" OR "conflicting task" OR "task prioritisation" OR "inattentional blindness" OR “task complexity”  AND  "functional performance" OR "mobility test" OR "balance test" OR "gait analysis"  OR "step length"  OR "cadence"  OR "step count"  ORr "step width"  OR "stance time"  OR "swing time"  OR "single support time"  OR "stride time"  OR "stride width"  OR "stride length"  OR "gait line"  OR "maximum force forefoot"  OR "maximum force midfoot"  OR “maximum force heel”  OR “double support time”  OR “gait speed”  OR “stride speed”  OR “motion”  OR “movement”  OR “motor$”  OR “locomotion”  OR “walking”  OR “balance”  OR “posture” OR "postural control" OR “postural sway” OR “sway velocity” OR “entropy” OR “displacement” OR“cognitive”  OR “neurocognitive”  OR “cognition$”  OR “executive”  OR “processing”  OR “spatial”  OR “visuospatial”  OR “memory”  OR “reaction$”  OR “speed” OR  “decision-making” OR “mental” OR “attention” OR “cognitive-motor” OR “motor-cognitive” OR “reaction” OR “planning” OR inhibition  AND  "vestibular dysfunction" OR dizziness OR drowsiness OR sadness OR “pressure in head” OR sensitivity OR “neck pain” OR tenderness  OR “blurred vision*” OR migraine* OR headache OR migrainous OR “impaired vision” OR “ocular dysfunction” OR "cognitive fatigue" OR “mental fatigue” OR depression OR fear OR anxiety OR irritability OR fatigue OR insomnia OR sleep OR somnolence OR “confusion” OR “energy deficit”  AND "Quantitative study" OR "Mixed-methods study" OR "Cross-sectional study" OR "Cohort study" OR "Case-control study" OR "Observational study" OR “intervention study” OR “randomized controlled trial” OR “non-randomized study” |
| --- | --- |

Resume

Population/Setting

• Persistent Concussion

• Symptoms associated with Concussion

Intervention

• Dual tasks

Outcome

• Gait parameters

• balance parameters

• Functional Motors tasks performance and precision

Study Type

• RCT

• Cohort Studies

• Observational Studies

• Experimental Studies• Intervention Studies
